# Quantitative assessment of the asphericity of pretherapeutic FDG uptake as an independent predictor of outcome in NSCLC

**DOI:** 10.1186/1471-2407-14-896

**Published:** 2014-12-01

**Authors:** Ivayla Apostolova, Julian Rogasch, Ralph Buchert, Heinz Wertzel, H Jost Achenbach, Jens Schreiber, Sandra Riedel, Christian Furth, Alexandr Lougovski, Georg Schramm, Frank Hofheinz, Holger Amthauer, Ingo G Steffen

**Affiliations:** Clinic of Radiology and Nuclear Medicine, University Hospital, Otto-von-Guericke University Magdeburg, Leipziger Strasse 44, Magdeburg, Germany; Clinic of Nuclear Medicine, University Medical Center Charité, Berlin, Germany; Lung Clinic Lostau gGmbH, Lostau, Germany; Clinic of Pneumology, University Hospital, Otto-von-Guericke University Magdeburg, Magdeburg, Germany; Helmholtz-Center Dresden-Rossendorf, Dresden, Germany

**Keywords:** Non-small cell lung cancer, Prognostic value, FDG-PET, Heterogeneity, Asphericity, Solidity

## Abstract

**Background:**

The aim of the present study was to evaluate the predictive value of a novel quantitative measure for the spatial heterogeneity of FDG uptake, the asphericity (ASP) in patients with non-small cell lung cancer (NSCLC).

**Methods:**

FDG-PET/CT had been performed in 60 patients (15 women, 45 men; median age, 65.5 years) with newly diagnosed NSCLC prior to therapy. The FDG-PET image of the primary tumor was segmented using the ROVER 3D segmentation tool based on thresholding at the volume-reproducing intensity threshold after subtraction of local background. ASP was defined as the relative deviation of the tumor’s shape from a sphere. Univariate and multivariate Cox regression as well as Kaplan-Meier (KM) analysis and log-rank test with respect to overall (OAS) and progression-free survival (PFS) were performed for clinical variables, SUVmax/mean, metabolically active tumor volume (MTV), total lesion glycolysis (TLG), ASP and “solidity”, another measure of shape irregularity.

**Results:**

ASP, solidity and “primary surgical treatment” were significant independent predictors of PFS in multivariate Cox regression with binarized parameters (HR, 3.66; p < 0.001, HR, 2.11; p = 0.05 and HR, 2.09; p = 0.05), ASP and “primary surgical treatment” of OAS (HR, 3.19; p = 0.02 and HR, 3.78; p = 0.01, respectively). None of the other semi-quantitative PET parameters showed significant predictive value with respect to OAS or PFS. Kaplan-Meier analysis revealed a probability of 2-year PFS of 52% in patients with low ASP compared to 12% in patients with high ASP (p < 0.001). Furthermore, it showed a higher OAS rate in the case of low versus high ASP (1-year-OAS, 91% vs. 67%: p = 0.02).

**Conclusions:**

The novel parameter asphericity of pretherapeutic FDG uptake seems to provide better prognostic value for PFS and OAS in NCSLC compared to SUV, metabolic tumor volume, total lesion glycolysis and solidity.

## Background

Lung cancer is the leading cause of cancer death and the second most frequently diagnosed cancer [1]. The TNM classification is accepted as the standard for therapy stratification [2]. Tumor staging based on the TNM classification is also known to be a strong predictor of prognosis [2]. Age, race, gender, tumor size, histology, and grade have also been shown to be independent predictors of survival [3]. Initial staging in patients with newly diagnosed NSCLC is needed to select the most appropriate therapeutic strategy and to determine prognosis. Combined positron emission tomography/computed tomography (PET/CT) using the tracer F-18-fluorodeoxyglucose (FDG) has been reported to be superior to conventional imaging modalities including CT and MRI in cancer staging especially for detection of nodal and metastatic site involvement [4]. By providing metabolic tumor characterization beyond clinical and structural information [5], FDG-PET has the potential to contribute independently to improved prediction of responsiveness or resistance to a specific treatment associated with in-vivo tumor biology.

Several studies suggest that high FDG uptake in the primary tumor at initial staging, mainly characterized by standardized uptake value (SUV), is associated with worse outcome in patients suffering from NSCLC [6–9]. Other studies propose the metabolic tumor volume (MTV) as a prognostic factor of disease recurrence and survival [10]. However, there are also studies in which the prognostic value of both these measures at initial staging is found to be unsatisfactory in NSCLC [11–14].

There is increasing recognition that the heterogeneity of pretherapeutic FDG uptake in the primary tumor can provide predictive information in several solid tumors [15, 16]. Quantifying the heterogeneity of FDG uptake appears promising for the prediction of therapy outcome as it might reflect the biological variability causing this intratumoral heterogeneity [17].

An increasing number of different measures have been proposed to quantify the voxel-wise or shape heterogeneity of tracer uptake [16, 18–20]. Some previous studies show encouraging results in prediction of treatment outcome from pretherapeutic FDG-PET, based on heterogeneity of uptake characterized by textural features in different carcinomas [16, 20]. Heterogeneity of FDG uptake, as measured by textural features, was shown to be a predictive factor also in patients with NSCLC [21, 22]. A measure of shape irregularity of the tumor’s FDG uptake was proposed by Eary et al. and has been shown to be associated with overall survival in certain types of sarcoma [18].

We were able to show that ‘asphericity’ (ASP), as a parameter for quantification of the spatial irregularity of FDG uptake, is a promising prognostic factor for tumor progression and outcome in patients with primary head and neck cancer [15]. The aim of this study was to evaluate the independent prognostic value of ASP in patients with NSCLC prior to therapy with respect to progression-free (PFS) and overall survival (OAS) in addition to conventional quantitative PET parameters such as SUVmax, SUVmean, metabolic tumor volume (MTV) and total lesion glycolysis (TLG) as well as relevant clinical parameters. Additionally, we compared ASP in terms of prognostic significance to another, previously described measure for quantitative characterization of shape irregularity of FDG uptake, the so-called “solidity” [19].

## Methods

### Patients

Patients were included retrospectively from our PET/CT database from February 2011 to July 2013 according to the following inclusion criteria: (i) patients had been referred for whole-body FDG-PET for staging of NSCLC prior to treatment, (ii) NSCLC was proven histologically, (iii) the primary tumor was clearly visible in the FDG-PET, (iv) histopathology and/or clinical/radiological follow-up of at least 12 months was available, (v) patients were treated with curative intent, (vi) the primary tumor measured at least 3 ml (the approximate lesion size that can be reliably delineated with the used delineation algorithm [23]). Patients in advanced stages with distant metastases (UICC stage IV) and patients treated with palliative intent were excluded from the analysis. This resulted in the inclusion of 60 patients (15 women, 45 men; mean age, 65.1 ± 9.5 years; median, 65.5 years; range, 45.9 - 80.6 years). Thirty-four of the tumors were adenocarcinomas, 23 were squamous cell carcinomas, one was a large-cell lung carcinoma and in 2 patients no histological subclassification was possible. Patient characteristics are summarized in Table 1. Tumor progression was defined by the follow-up as occurrence of local or regional recurrence, local tumor progression, distant metastases or a combination of these.

**Table 1:** **Patient characteristics**

Variable	Number (%)
Total	60 (100)
Gender	
Male	45 (75)
Female	15 (25)
T stage (TNM)	
1	13 (22)
2	26 (43)
3	16 (27)
4	5 (8)
UICC stage	
I	10 (17)
II	17 (28)
III	33 (55)
IIIA	25 (42)
IIIB	8 (13)
Histology	
Adenocarcinoma	34 (57)
Squamous cell cancer	23 (38)
NSCLC, other	3 (5)
Localization	
Central	28 (47)
Peripheral	32 (53)
Therapy	
Surgery	39 (65)
Surgery only	11 (18)
Surgery + CTx	14 (23)
Surgery + RCTx	14 (23)
Primary RCTx	21 (35)

CTx = chemotherapy; RTx = radiotherapy; RCTx = radiochemotherapy.

The study protocol had been approved by the Ethics Committee of the University Hospital Magdeburg A. ö. R. at the Otto-von-Guericke University (reference number, 159/13; RAD233) and complied with the Declaration of Helsinki.

### PET imaging

Patients received a whole-body PET/CT examination with 18-F-FDG (Biograph mCT 64, Siemens Medical, Erlangen, Germany). The PET protocol included a fasting period of at least 8 h followed by confirmation of a blood glucose level ≤150 mg/dl prior to the scanning procedure. PET scans were performed at a median of 64.2 min (IQR, 62.2 - 69.9 min) after intravenous injection of 179 to 254 MBq (median, 235 MBq) of FDG. Whole-body imaging was performed from base of the skull to the proximal femora (5–7 bed positions; emission (each), 3 minutes). PET images were derived from a 200 × 200 acquisition matrix and were iteratively reconstructed with scatter correction using Siemens ultraHD-PET algorithm (2 iterations, 21 subsets). The algorithm uses time-of-flight (TOF) analysis and accounts for the point spread function (PSF) of the specific scanner (Siemens® Healthcare, Erlangen, Germany). An attenuation map was generated from the whole-body low-dose CT (50 mAs/120 kV; detector collimation, 16 × 1.2 mm; exposure time, 0.5 s; spiral pitch factor, 0.8) reconstructed with a slice thickness of 5 mm (matrix size, 512 × 512; voxel size, 1.5 × 1.5 × 5.0 mm).

### Image analysis

The metabolically active part of the tumor was delineated by an automatic algorithm based on adaptive thresholding, taking the local background into account [23]. VOI definition and VOI analysis were performed by two observers in consensus to fully include the primary tumor and exclude neighboring tissues using the software ROVER (ABX, advanced biochemical compounds GmbH, Radeberg, Germany). Detailed description of the algorithm was published elsewhere [23, 24]. The result of the automatic delineation was inspected visually and corrected manually if non-tumor parts were included in the segmentation volume. The ASP of the resulting volume of interest (VOI) was computed together with SUVmax, SUVmean, the metabolic tumor volume (MTV = VOI volume) and the total lesion glycolysis (TLG = MTV * SUVmean) [15] The SUV was calculated with respect to total body weight according to the formula: SUV = tracer concentration in tissue (MBq/ml)/injected dose (MBq) × total body weight (kg). Both, tracer concentration in tissue and injected dose were decay corrected to the start time of the PET emission scan.

### Asphericity (ASP)

The ASP of the primary tumor was defined as:


where S and V are the surface and volume of the MTV, respectively.

**Figure 1:**
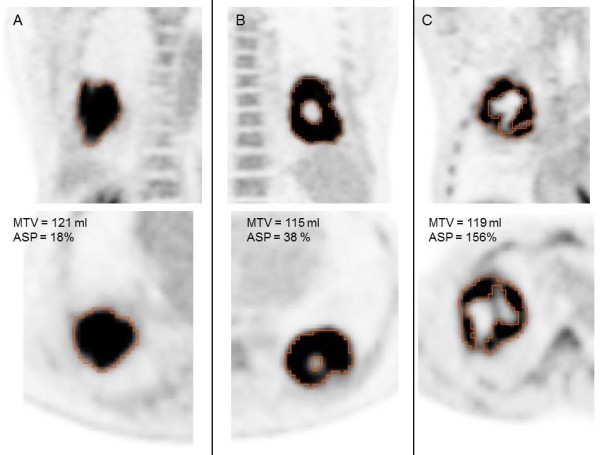
**Orthogonal images of three representative examples of tumors with comparable MTV (range, 115–121 ml) but different ASP values: (A) 18%, (B) 38% and (C) 156%.** Segmentation volume of the metabolically active tumor indicated by red line.

The rationale for this definition is described in detail in a recent publication of our group [15]. ASP is independent of the lesion size. It is zero for spherical lesions and is larger than zero for all other lesion types. ASP = 0.5 = 50%, for example, means that the surface of the lesion is 50% larger than the surface of a sphere with the same volume. Thus, ASP is a quantitative measure of shape irregularity caused by necrotic tumor parts or invasive growth. Figure 1 shows orthogonal slices of three examples.

### Solidity

For comparison of ASP with another published measure of spatial irregularity, we included the solidity in our analysis. For computation of solidity, we followed the description of el Naqa et al. [19], where solidity is defined as the proportion of voxels inside the convex hull of the ROI which are also inside the ROI itself. The convex hull was computed with the geometry package of R language and environment for statistical computing version 3.0.2, which uses the QHull algorithm [24]. The remaining ROI analyses were performed with ROVER version 2.1.20 (ABX, Radeberg, Germany).

#### Statistical analysis

Data were analyzed using the R software (Version 2.15.3, The R Foundation for Statistical Computing, Vienna, Austria, http://www.R-project.org). Non-parametric distribution of parameters was assumed for histograms and Q-Q plots. Median and interquartile ranges (IQR) were therefore used as descriptives. The correlation of metric variables was tested by the Spearman’s rank correlation method and illustrated by scatter plots.

The association of PFS and OAS with all clinically relevant parameters (gender, histology, tumor stage (T3/T4 *vs.* T1/T2), UICC stage (III *vs.* I/II), primary tumor localization (central *vs.* peripheral), different treatment strategies), as well as all quantitative PET parameters were analyzed using univariate Cox proportional-hazards regression, in which the PET parameters were included as metric values. Additionally, metric parameters were binarized using cut-offs. The thresholds for survival analysis were not determined by ROC analysis, as this method does not consider survival times and censored data. Optimal thresholds were therefore calculated by performing univariate Cox regression for each measured data value and the threshold leading to the hazard ratio with the highest significance was taken as optimal cut-off. In order to avoid too small group sizes only data values within the interquartile range were considered as optimal cut-off. The impact of the resulting binarized parameters on PFS and OAS was analyzed using univariate Cox regression, Kaplan-Meier curves and log-rank test. Furthermore, the predictive value of ASP was analyzed in multivariate Cox regression including parameters which showed a tendency to significance (p ≤ 0.10) in the univariate analysis (MTV, surgery and ASP). TLG was excluded from multivariate analysis due to high collinearity with MTV. Statistical significance was assumed at a p-value of less or equal to 0.05.

## Results

### Patient outcome

Patients had an overall survival rate of 73.3% with a median OAS of survivors of 20.0 months (IQR, 15.5 - 24.3 months). Sixteen patients died after a median time of 10.2 months (IQR, 7.3 - 15.6). Recurrence or progression occurred in 29 patients after a median time period of 8.9 months (IQR, 5.5 - 14.0 months).

### Quantitative PET parameters

**Figure 2:**
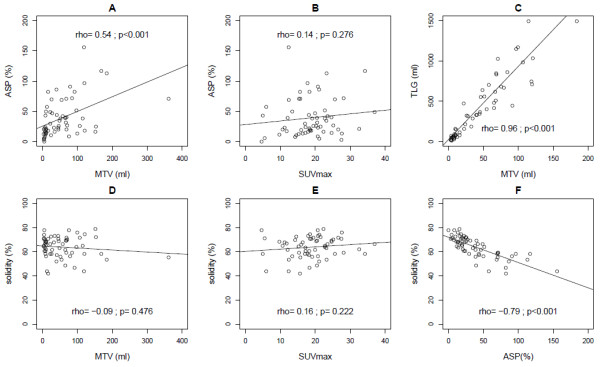
**Correlations between ASP and MTV (A), ASP and SUVmax (B) and TLG and MTV (C), solidity and MTV (D), solidity and SUVmax (E), solidity and ASP (F).**

Descriptive values of SUVmax, SUVmean, MTV, TLG, ASP and solidity are given in Table 2. TLG and MTV were strongly correlated (rho = 0.96, p < 0.001). There was a moderate correlation between ASP and MTV (rho = 0.54, p < 0.001) and no significant correlation between ASP and SUVmax (Figure 2, A-B). Solidity was significantly inversely correlated with ASP (rho = -0.79, p < 0.001) but not with SUVmax and MTV (Figure 2, D-F)

**Table 2:** **Quantitative PET parameters**

Parameter	Value
SUVmax	
Median	18.7
IQR	15.3 - 22.7
Range	4.6 - 37.0
SUVmean	
Median	8.5
IQR	6.9 - 11.8
Range	3.0 - 21.8
MTV (ml)	
Median	42.7
IQR	10.0 - 76.5
Range	3.2 - 361.7
TLG (ml)	
Median	355.5
IQR	81.3 - 718.6
Range	14.2 - 2980.9
ASP (%)	
Median	26.3
IQR	16.5 - 50.5
Range	0.2 - 155.8
Solidity	
Median	64.9
IQR	58.2 – 70.6
Range	41.9 – 78.7

Median, IQR and range of SUVmax, SUVmean, metabolic tumor volume (MTV), total lesion glycolysis (TLG), ASP and solidity.

### PFS

**Figure 3:**
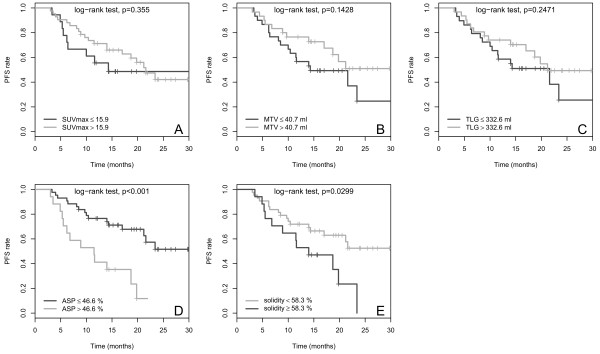
**Kaplan-Meier curves for the quantitative PET parameters SUVmax (A), MTV (B), TLG (C), ASP (D) and solidity (E) with respect to PFS.** Cut-off values and p-values are shown on each panel.

The results of the univariate Cox regression with respect to PFS of the PET parameters as metric variables are shown in Table 3. There was a significant effect for ASP (p < 0.01) but neither for SUVmax, SUVmean, MTV, TLG or solidity, nor for the clinical parameters. However, a tendency to significance was observed for primary surgical treatment (with vs. without, HR, 1.9; p = 0.09). After binarization of metric parameters the univariate Cox regression showed a significant effect of ASP (cut-off, 46.6%) with an HR of 3.4 (p = 0.001; Table 4) whereas no significant effect was seen with conventional semi-quantitative PET parameters. Solidity, however, showed a significant effect after binarization (HR, 2.2; p = 0.03, cut-off 58.3). Multivariate Cox regression including binarized ASP and “primary surgical treatment” revealed an HR of 3.7 (p < 0.001) for high ASP, and an HR of 2.1 (p = 0.05) for “no primary surgery”. Multivariate Cox regression with solidity and “primary surgical treatment” as input parameters included only solidity (HR, 2.11; p = 0.05) in the final model. Kaplan-Meier curves for PFS in association with binarized SUVmax, MTV, TLG, ASP and solidity are shown in Figure 3.

**Table 3:** **Univariate Cox proportional-hazards regression (metric variables) with respect to PFS and OAS**

Variable		PFS			OAS	
	HR	95%-CI	p-value	HR	95%-CI	p-value
SUVmax	1.00	0.94 - 1.06	0.94	0.97	0.91 - 1.05	0.48
SUVmean	0.99	0.88 - 1.10	0.80	0.91	0.78 - 1.06	0.21
MTV	1.00	1.00 - 1.01	0.49	1.01	1.00 - 1.01	0.07
TLG	1.00	1.00 - 1.00	0.89	1.00	1.00 - 1.00	0.21
ASP	1.01	1.00 - 1.03	**0.009**	1.02	1.00 - 1.03	**0.03**
Solidity	0.97	0.93 - 1.01	0.12	0.98	0.92 – 1.03	0.39
Gender: female	1.05	0.44 - 2.46	0.92	0.44	0.10 - 1.95	0.28
Histology: adenocarcinoma	0.90	0.21 - 3.90	0.89	1.04	0.13 - 8.17	0.97
Histology: SCC	0.44	0.09 - 2.07	0.30	0.70	0.08 - 5.98	0.74
Localization: central	0.68	0.31 - 1.47	0.33	2.23	0.81 - 6.10	0.12
T stage (TNM): T3/T4	0.71	0.31 - 1.61	0.41	1.24	0.45 - 3.42	0.68
UICC stage: III A-B	0.97	0.47 - 2.01	0.93	2.10	0.73 - 6.05	0.17
Surgery: no	1.90	0.91 - 3.97	0.09	3.56	1.29 - 9.83	**0.01**
Radiotherapy: no	0.66	0.31 - 1.41	0.28	0.40	0.13 - 1.26	0.12
Chemotherapy: no	0.66	0.23 - 1.91	0.45	0.59	0.13 - 2.60	0.49

The respective hazard ratio (HR), 95%-confidence interval (95%-CI) and p-value are displayed. SCC = squamous cell cancer; RTx = radiotherapy; CTx = chemotherapy; RCTx = radiochemotherapy.

Significant p-values are indicated by bold numbers.

**Table 4:** **Results of univariate Cox regression for binarized quantitative PET parameters**

Variable		PFS				OAS		
	Cut-off	HR	95%-CI	p-value	Cut-off	HR	95%-CI	p-value
SUVmax	>15.9	0.69	0.31 - 1.53	0.36	>17.2	0.44	0.16 - 1.19	0.11
SUVmean	>8.4	0.57	0.27 - 1.20	0.14	>9.0	0.41	0.14 - 1.19	0.10
MTV (ml)	>40.7	0.58	0.27 - 1.21	0.15	>75.5	1.78	0.64 - 4.90	0.27
TLG (ml)	>332.6	0.65	0.31 - 1.36	0.25	>82.5	0.48	0.18 - 1.29	0.14
ASP (%)	>46.6	3.44	1.61 - 7.33	**0.001**	>50.2	2.97	1.10 - 8.00	**0.03**
Solidity	<58.3	2.24	1.25 - 5.58	**0.03**	<66.8	2.42	0.78 - 7.52	1.12

Significant p-values are indicated by bold numbers.

### OAS

**Figure 4:**
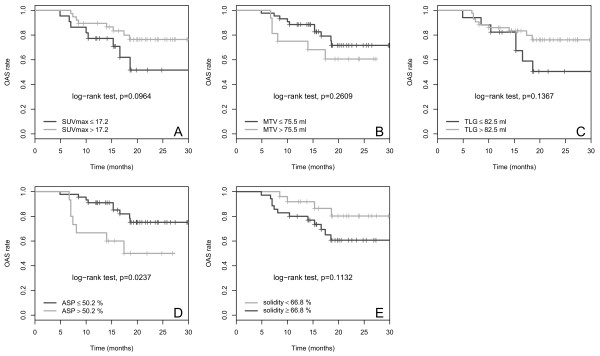
**Kaplan-Meier curves for the quantitative PET parameters SUVmax (A), MTV (B), TLG (C), ASP (D) and solidity (E) with respect to OAS.** Cut-off values and p-values are shown on each panel.

The results of univariate Cox regression with respect to OAS for the metric variables are summarized in Table 3. A significant effect was observed only for ASP (p = 0.03) and “primary surgical treatment” (p = 0.01), while a tendency towards significance was seen for MTV (p = 0.07) and the treatment combination surgery + CTx (HR, 2.8; p = 0.07). In univariate Cox regression analysis including binarized parameters an HR of 3.0 (p = 0.03) was observed for ASP (cut-off, 50.2%) whereas other semi-quantitative parameters showed no significant effect (Table 4). Multivariate Cox regression analysis with ASP and “primary surgical treatment” included both parameters in the final model for prediction of OAS (ASP: HR, 3.2; p = 0.02; “no surgery”: HR, 3.8; p = 0.01). Multivariate Cox regression analysis with MTV and “primary surgical treatment” as input parameters included only surgical treatment (HR, 4.0; p = 0.008) but not MTV (HR, 2.2; p = 0.13) in the final model. Multivariate analysis with surgical treatment and solidity as input parameters also included only surgical treatment in the final model (HR, 3.7; p = 0.01). Kaplan-Meier curves with respect to OAS for binarized SUVmax, MTV, TLG, ASP and solidity are depicted in Figure 4.

## Discussion

High heterogeneity of tumors with respect to various biological parameters is known to be associated with aggressive tumor behavior, response to therapy and survival in a number of cancer types [17, 25]. This is the rationale for the quantitative evaluation of the heterogeneity of the FDG-PET uptake in tumor lesions, which is assumed to capture the heterogeneity of tumor biology to some extent, although the exact relationship has not yet been fully elucidated. FDG-PET based heterogeneity measures have been found to be superior to tumor volume and conventional FDG-PET based measures including SUVs, MTV and TLG for various indications [16, 18, 19].

In the present study we have demonstrated that relevant improvement of outcome prediction in patients with NSCLC treated with curative intent can be achieved using ASP, a novel parameter for quantitative characterization of the shape irregularity of the FDG uptake in the primary tumor. In curatively treated patients, binarized ASP was an independent significant prognostic factor for both PFS (HR, 3.4; p = 0.001) and OAS (HR, 2.97; p = 0.03) as well as ‘primary surgical treatment’ (PFS: HR, 2.09; p = 0.05 and OAS: HR, 3.78; p = 0.01). The probability of 2-years PFS decreased from 52% in the patients with low ASP (≤ 46.6%) to 12% in the patients with high ASP (> 46.6%). A similar, significant effect was observed for OAS where 1-year OAS decreased from 91% to 67% in patients with high ASP (> 50.2%).

A pilot study of the novel parameter suggested that ASP is a strong independent predictor of outcome in patients with primary manifestation of head and neck cancer. Univariate Cox regression revealed hazard ratios of 7.8 and 7.4 for PFS and OAS, respectively [15]. The hazard ratios associated with high ASP were somewhat lower in the NSCLC patient group of the present study.

In head and neck cancer we found that combining ASP with the MTV further improved the predictive power (HR, 22.7 for PFS and 13.2 for OAS), despite a moderate correlation between MTV and ASP similar to the correlation between MTV and ASP in the present study (rho = 0.54, Figure 2A). The factors that mediate a positive correlation between ASP and MTV include spatial resolution and necrosis. Limited spatial resolution of PET imaging causes small lesions to appear more spherical (lower ASP) than they actually might be. Necrosis, which results in increased ASP by producing additional internal surface area within the MTV (Figure 1), is more likely to occur in large tumors than in small ones. However, the fact that the correlation between ASP and MTV was only moderate suggests that the two parameters are not redundant. Nevertheless, in the present study the combination of ASP and MTV did not improve the predictive power over ASP alone (data not shown). This accords with the fact that MTV provides weaker prognostic power in NSCLC in comparison to head and neck cancer [15].

None of the conventional PET metabolic parameters, including SUVmax, SUVmean, MTV and TLG, showed a significant predictive effect in the current study. Previous studies of the prognostic value of SUVs in NSCLC reported rather variable results. A few studies reported an association between high SUV and poor prognosis in early stage [6–8] as well as locally advanced stage NSCLC [9]. However, other studies did not support this finding [11, 12]. For example, a large multicenter study including 250 patients treated with primary radiochemotherapy found no significant prognostic value of *pre*treatment SUVs; only *post*treatment SUVs were found to be associated with survival [12]. The level of evidence is similar for the volumetric PET parameters MTV and TLG. Most published studies report an association between survival and these volumetric PET parameters [10, 26–28], however, contradictory results have also been published [13, 14]. For example, Soussan et al. found only the post- to pretreatment difference of volumetric PET parameters to be predictive of outcome in patients with stage III NSCLC; pretreatment parameters alone were not predictive. The somewhat conflicting results on the prognostic value of conventional pretreatment FDG-PET measures in the literature suggest that their prognostic value is rather limited and therefore underline the importance of identifying novel PET parameters which provide stronger predictive power for risk stratification from the baseline PET prior to therapy.

Concerning clinical parameters, previous studies have demonstrated the prognostic value of both patient characteristics, e.g. age or ECOG performance status [3], and tumor specific characteristics, e.g. tumor stage, histological differentiation, blood vessel infiltration, lymph vessel infiltration and biological markers [29]. In the present study, only one of the clinical parameters considered, primary treatment strategy, reached the level of statistical significance as prognostic factor. This might be due to the fact that the sample size did not provide sufficient statistical power to detect such effects. This suggests that the prognostic value of these clinical variables is lower than the prognostic value of the ASP in pretreatment FDG-PET. Having undergone primary surgical treatment was a predictor of outcome in the present study, particularly with respect to OAS. This is in accordance with the results of large therapy trials [30].

There are two recent studies on the use of textural heterogeneity measures in NSCLC. Cook et al. demonstrated that high heterogeneity of FDG uptake in the primary tumor, as characterized by textural features, was associated with non-response to chemoradiotherapy and poor prognosis. In agreement with the present study neither SUVs nor volumetric parameters were found to be predictive [21]. Tixier and co-workers compared a visual score of heterogeneity with quantitative heterogeneity measures based on textural analysis in a mixed population of lung cancer patients (n = 102) [22]. The authors found that the visual score correlated with the quantitative heterogeneity measures, but only the quantitative measure provided independent prognostic value. This finding supports the use of quantitative measures, which are determined either fully or semi-automatically and are therefore not limited by intra- and inter-reader variability. In addition, the continuous range of values of quantitative measures most probably allows for better discrimination than visual scores with only a few discrete values. Beside textural features, several other parameters were found to be predictive for OAS (but not PFS) in the study of Tixier et al. These were SUVmean and MTV, as well as surgical treatment; the latter in agreement with the present study.

The textural features used by Cook et al. and Tixier et al. mainly characterize the voxel-wise heterogeneity of the FDG uptake within the tumor. In contrast, ASP is a quantitative measure of the irregularity of the 3-dimensional contour of the tumor in its FDG-PET image, i.e. the ASP depends on the shape of the surface of the metabolically active volume, but it is not sensitive to the variability of the FDG uptake in interior tumor voxels. The shape-based measure ASP therefore depicts different information on tumor heterogeneity to textural features based on voxel-by-voxel variability of tracer uptake. An advantage of ASP compared to other measures of heterogeneity is that it is easily computed (just by counting voxels) from the ROI which delineates the metabolically active part of the tumor, since only the volume and the surface area of this ROI are required. Thus, computation of ASP is easily integrated in any existing software which provides a ROI tool [15]. To the best of our knowledge, the present study is the first to evaluate the prognostic value of the shape irregularity of FDG uptake in NSCLC.

We compared ASP to another shape-based measure of heterogeneity, the parameter “solidity” proposed by El Naqa and co-workers for quantitative characterization of the convexity of the metabolically active tumor lesion [19]. These authors identified solidity as a promising prognostic parameter in cervical as well as head and neck cancer. In ROC analyses solidity provided a larger area under the curve than SUVmax and some uptake-based textural features [19]. In the present study, solidity was found to be inversely correlated with ASP, as was to be expected. However, in contrast to ASP, solidity did not provide significant prognostic information according to univariate Cox regression, either for PFS or for OAS. Only after binarization did solidity show a significant association with PFS (not OAS), and this was still considerably lower than that found for ASP (HR 2.2 versus 3.4), although the cut-off was optimized independently for both parameters. The higher prognostic value of ASP compared to solidity was confirmed by Kaplan-Meier and multivariate regression analyses.

Limitations of the present study are its retrospective character, the inclusion of patients from a single institution only, as well as the limited sample size. Our results must therefore still be considered as preliminary. We have initiated a prospective trial to confirm the prognostic value of ASP in a larger cohort of curatively treated patients with NSCLC and to prospectively test the cut-offs proposed in the present study. If the strong prognostic power of ASP is confirmed in this study, the clinical value of ASP for stratification of high risk patients to intensified primary chemoradiation of locally advanced NSCLC will be evaluated in further studies.

## Conclusions

The novel parameter asphericity of pretherapeutic FDG uptake seems to provide a better prognostic value for PFS and OAS in NCSLC than SUV, metabolic tumor volume, total lesion glycolysis or the previously described shape feature, solidity.

## Consent

Written informed consent was obtained from all patients for the publication of this report and any accompanying images.
